# Evolution and codon usage bias of mitochondrial and nuclear genomes in *Aspergillus* section *Flavi*

**DOI:** 10.1093/g3journal/jkac285

**Published:** 2022-10-28

**Authors:** Miya Hugaboom, Elizabeth Anne Hatmaker, Abigail L LaBella, Antonis Rokas

**Affiliations:** Department of Biological Sciences, Vanderbilt University, Nashville, TN 37235, USA; Department of Biological Sciences, Vanderbilt University, Nashville, TN 37235, USA; Evolutionary Studies Initiative, Vanderbilt University, Nashville, TN 37235, USA; Department of Bioinformatics and Genomics, University of North Carolina at Charlotte, Charlotte, NC 28223, USA; Department of Biological Sciences, Vanderbilt University, Nashville, TN 37235, USA; Evolutionary Studies Initiative, Vanderbilt University, Nashville, TN 37235, USA

**Keywords:** mitogenome, phylogeny, codon usage bias, *Aspergillus*, section *Flavi*, fungi

## Abstract

The fungal genus *Aspergillus* contains a diversity of species divided into taxonomic sections of closely related species. Section *Flavi* contains 33 species, many of industrial, agricultural, or medical relevance. Here, we analyze the mitochondrial genomes (mitogenomes) of 20 *Flavi* species—including 18 newly assembled mitogenomes—and compare their evolutionary history and codon usage bias patterns to their nuclear counterparts. Codon usage bias refers to variable frequencies of synonymous codons in coding DNA and is shaped by a balance of neutral processes and natural selection. All mitogenomes were circular DNA molecules with highly conserved gene content and order. As expected, genomic content, including GC content, and genome size differed greatly between mitochondrial and nuclear genomes. Phylogenetic analysis based on 14 concatenated mitochondrial genes predicted evolutionary relationships largely consistent with those predicted by a phylogeny constructed from 2,422 nuclear genes. Comparing similarities in interspecies patterns of codon usage bias between mitochondrial and nuclear genomes showed that species grouped differently by patterns of codon usage bias depending on whether analyses were performed using mitochondrial or nuclear relative synonymous usage values. We found that patterns of codon usage bias at gene level are more similar between mitogenomes of different species than the mitogenome and nuclear genome of the same species. Finally, we inferred that, although most genes—both nuclear and mitochondrial—deviated from the neutral expectation for codon usage, mitogenomes were not under translational selection while nuclear genomes were under moderate translational selection. These results contribute to the study of mitochondrial genome evolution in filamentous fungi.

## Introduction

The fungal genus *Aspergillus* is an important genus of filamentous fungi. The genus houses species with industrial applications, important pathogens of humans, animals, and crops, producers of potent carcinogenic mycotoxins, and the genetic model organism *Aspergillus nidulans* (de Vries *et al.* 2017). *Aspergillus* is divided into taxonomic sections of closely related species. Section *Flavi* consists of 33 species, many of which have industrial, agricultural, or medical relevance (Gourama and Bullerman 1995; Hedayati *et al.* 2007; de Vries *et al.* 2017; Frisvad *et al.* 2018; Homa *et al.* 2019). For example, *Aspergillus oryzae* constitutes an important cell factory for enzyme production and, along with *Aspergillus sojae*, is vital to the production of a range of fermented foods (Machida *et al.* 2008; Sato *et al.* 2011). Conversely, *Aspergillus flavus* is an effective producer of aflatoxin B, a potent carcinogenic mycotoxin, and has been found to be both a plant contaminant and occasional pathogen, as well as an opportunistic human pathogen (Hedayati *et al.* 2007; Hoffmeister and Keller 2007; Dolezal *et al.* 2014). To better understand the diversity of these fungi, a recent study sequenced the genomes for 23 of the 33 known *Flavi* species to gain insights into their biology and evolution (Kjærbølling *et al.* 2020).

Previous genomic analyses of section *Flavi* focus almost exclusively on the nuclear genomes of the sequenced species (de Vries *et al.* 2017; Kjærbølling *et al.* 2020); the sole exception was a 2012 study that described the genomes of 6 diverse *Aspergillus* species, including 2 from section *Flavi* (Joardar *et al.* 2012). However, whole genome sequencing captures nucleotide sequences from both nuclear and organellar genomes. Fungal mitochondria have been linked to diverse processes including energy metabolism, cell differentiation, drug resistance, biofilm and hyphal growth regulation, and virulence, among others (Sanglard *et al.* 2001; Burger *et al.* 2003; Martins *et al.* 2011; Chatre and Ricchetti 2014; Calderone *et al.* 2015). Using appropriate software, mitochondrial reads can be effectively filtered and separated from nuclear reads within existing whole-genome sequencing datasets to be used for mitochondrial genome (mitogenome) assembly and annotation (Hugaboom *et al.* 2021). Fungal mitogenomes, including those of *Aspergillus* species, are typically circular and composed of a single chromosome (Brown *et al.* 1985; Joardar *et al.* 2012). Mitogenomes replicate independently from the nuclear genome and cell cycle and tend to have high copy number. Fourteen protein-coding genes involved in the electron transport chain are highly conserved within fungal mitogenomes (Gray *et al.* 1999; Lavín *et al.* 2008; Joardar *et al.* 2012). Genes for 2 ribosomal rRNA subunits, 1 large and 1 small, and a variable number of tRNAs also tend to be housed in the mitogenome (Gray *et al.* 1999; Lavín *et al.* 2008; Joardar *et al.* 2012). Variation in fungal mitogenomes is largely due to differences in intron distribution and the variable presence of accessory mitochondrial genes, even between closely related species (Joardar *et al.* 2012; Li, Wang, *et al.* 2019; Li, Xiang, *et al.* 2019; Wang *et al.* 2020; Zhang *et al.* 2020; Chen *et al.* 2021). Importantly, mitogenomes also differ from nuclear genomes in their inheritance pattern. Although fungal mitogenomes are not always uniparentally inherited and can exhibit recombination (Basse 2010; Stein and Sia 2017; Zardoya 2020; Mukhopadhyay and Hausner 2021), *Aspergillus* mitogenomes are uniparentally inherited and rarely display recombination, offering a unique phylogenetic perspective (Coenen *et al.* 1996; Santamaria *et al.* 2009; Kjærbølling *et al.* 2020). Mitochondrial genomes may therefore hold clues to both the biology and evolution of these fungal species.

Another key difference between mitogenomes and nuclear genomes is codon usage bias (CUB). CUB refers to the different frequency of synonymous codons—those that code for the same amino acid—in coding DNA. Changes in synonymous codons do not alter primary protein sequence and were thus once assumed to be selectively neutral (Jia and Higgs 2008; Wei *et al.* 2014; LaBella *et al.* 2019). However, CUB has been found to influence numerous cellular processes, particularly those associated with translation (Stoletzki and Eyre-Walker 2007; Zhou *et al.* 2009). This is hypothesized to be due to codon optimization: the tendency for codon usage to be correlated to the abundance of tRNA molecules in the genome (Post *et al.* 1979; Nakamura *et al.* 1980; Ikemura 1981; Gouy and Gautier 1982; Sharp and Li 1986a; Thomas *et al.* 1988). During translation, mRNAs containing optimized codons—codons corresponding to the tRNA pool of the cell—are translated more efficiently than those with nonoptimal codon usage (Bulmer 1991; Xia 1998; Chevance *et al.* 2014; Presnyak *et al.* 2015; Hanson and Coller 2018). In many organisms, this leads to a correlation between codon usage and protein production (Ikemura 1981; Bulmer 1991; Gustafsson *et al.* 2004; Hiraoka *et al.* 2009; Roymondal *et al.* 2009; Zhou*et al*. 2016; Payne and Alvarez-Ponce 2019; Sahoo *et al.* 2019). Importantly, mitogenomes house their own set of tRNAs that is distinct from that of the nuclear genome and thus may exhibit patterns of CUB shaped by optimization to a greater extent by the mitochondrial set of tRNAs (tRNAome) than the nuclear tRNAome. Variation in synonymous codon usage is a widespread phenomenon at codon, gene, and whole genome levels in nuclear and mitochondrial genomes (LaBella *et al.* 2019; LaBella *et al.* 2021; Wint *et al.* 2022). This variation in codon usage likely reflects a balance of mutational bias (e.g. GC content), natural selection (e.g. translational selection), and genetic drift (Ikemura 1985; Shields and Sharp 1987; Sharp *et al.* 1993; Wei *et al.* 2014). The balance of these forces varies between organisms. In many microbes, for example, translational selection plays a large role, whereas mutational bias plays the primary role in humans (Sharp *et al.* 1993). However, analysis of mitochondrial CUB in fungi is limited (Kamatani and Yamamoto 2007; Carullo and Xia 2008). Understanding patterns of CUB can provide insight into the evolutionary history of individual genes and entire genomes.

To gain insights into the evolution of mitogenomes from the section *Flavi*, we analyzed mitochondrial genomes of 20 section *Flavi* species—including 18 newly assembled ones—and compared their phylogeny and CUB to the nuclear genomes of the same species. All mitogenomes were confirmed to be circular DNA molecules of low GC content with highly conserved gene content and gene order. Genomic content and size differed greatly between mitochondrial and nuclear genomes. We then inferred and compared phylogenies constructed from mitochondrial vs nuclear genes. The presence and high copy number of mitogenomes within the cell as well as the lack of recombination relative to nuclear genomes (for a discussion of fungal mitochondrial genome recombination, see Zardoya 2020; Mukhopadhyay and Hausner 2021; Stein and Sia 2017) make mitochondrial genes and genomes useful markers for phylogenetic analyses. Currently, phylogenies constructed for *Aspergillus* section *Flavi* are based solely on nuclear genome markers (Kjærbølling *et al.* 2020; Shen *et al.* 2020). Phylogenetic analysis based on 14 concatenated mitochondrial genes (mitogenes) predicted evolutionary relationships largely consistent with those inferred by a phylogeny based on nuclear data. We then investigated CUB in mitochondrial and nuclear genomes. At the gene-level, we found that patterns of CUB reflect whether the gene is mitochondrial or nuclear in origin as well as mitogene identity rather than species of origin; these patterns were influenced largely by GC content of the third codon position. Finally, we determined that although most genes—both nuclear and mitochondrial—deviated from the neutral expectation, mitogenomes were not under translational selection while nuclear genomes were under moderate translational selection. By providing mitogenome assemblies for 20 section *Flavi* species and comparing the evolution of mitochondrial and nuclear genes in section *Flavi*, our study advances our understanding of genome evolution in the genus *Aspergillus*.

## Methods

### Genomic data

We used strains from 21 species within section *Flavi* and *Aspergillus niger* (section *Nigri*) as an outgroup for phylogenetic analyses. For the mitochondrial dataset, we used a combination of available mitochondrial reference genomes and newly assembled whole-genome sequencing reads. Three previously assembled mitochondrial reference genomes (*A. sojae*, *A. oryzae*, and *A. niger*) were downloaded from NCBI’s Nucleotide Database (Juhász *et al.* 2008; Machida *et al.* 2008; Sato *et al.* 2011). For new assemblies, previously sequenced paired-end Illumina whole genome sequence reads were downloaded from NCBI’s Sequence Read Archive (Kjærbølling *et al.* 2020; Hatmaker *et al.* 2022).

Annotated protein-coding nucleotide sequences (CDS) for each nuclear genome were downloaded from JGI MycoCosm (Grigoriev *et al.* 2014; Kjærbølling *et al.* 2020) except for *A. sojae*, *A. flavus*, and *Aspergillus nomiae.* For *A. sojae*, strain-matched nuclear annotations were not available and, thus, this species was not included in phylogenetic inferences or any analyses based on nuclear genomic data. For *A. flavus* and *A. nomiae*, we used annotations from recently assembled genomes (Hatmaker *et al.* 2022), extracting the CDS regions. Strains and data sources are summarized in Table 1.

**Table 1.** Summary of sources of sequencing data.

Species	SRA accession/NCBI reference sequence GenBank	Source
*Aspergillus flavus*	SRR18725159	Hatmaker *et al.* (2022)
*Aspergillus transmontanesis*	SRR8398939	Kjærbølling *et al.* (2020)
*Aspergillus arachidicola*	SRR8398876	Kjærbølling *et al.* (2020)
*Aspergillus nomiae*	SRR19369914	Hatmaker *et al.* (2022)
*Aspergillus parasiticus*	SRR8840397	Kjærbølling *et al.* (2020)
*Aspergillus sergii*	SRR8840616	Kjærbølling *et al.* (2020)
*Aspergillus sojae*	AP014506.1	Sato *et al.* (2011)
*Aspergillus oryzae*	NC_008282.1	Machida *et al.* (2005)
*Aspergillus minisclerotigenes*	SRR8398929	Kjærbølling *et al.* (2020)
*Aspergillus caelatus*	SRR8840396	Kjærbølling *et al.* (2020)
*Aspergillus pseudocaelatus*	SRR8840541	Kjærbølling *et al.* (2020)
*Aspergillus pseudotamarii*	SRR8840579	Kjærbølling *et al.* (2020)
*Apsergillus tamarii*	SRR8840604	Kjærbølling *et al.* (2020)
*Aspergillus pseudonomiae*	SRR8840540	Kjærbølling *et al.* (2020)
*Aspergillus bertholletius*	SRR8398880	Kjærbølling *et al.* (2020)
*Aspergillus alliaceus*	SRR8396970	Kjærbølling *et al.* (2020)
*Aspergillus coremiiformis*	SRR8398877	Kjærbølling *et al.* (2020)
*Aspergillus leporis*	SRR8398928	Kjærbølling *et al.* (2020)
*Aspergillus avenaceus*	SRR8839916	Kjærbølling *et al.* (2020)
*Aspergillus novoparasiticus*	SRR8398934	Kjærbølling *et al.* (2020)
*Aspergillus niger*	NC_007445.1	Juhász et al. (2008)

Reference mitochondrial genomes were used for *Aspergillus sojae*, *A. oryzae*, and *A. niger*. Raw paired-end whole genome sequencing reads were used for the remaining species.

### Mitochondrial genome assembly

Data from the whole genome sequence read files were extracted into usable format (FASTQ files) using SRA Toolkit v2.9.6-1 (Leinonen *et al.* 2011). Mitochondrial genomes were assembled from the raw reads of each species using the organelle genome assembler GetOrganelle v1.7.4.1 (Jin *et al.* 2020). Following the method of Hugaboom *et al.* (2021), we used the internal GetOrganelle fungal database (-F fungus_mt) and default parameter values for number of threads, extension, and k-mers to assemble the mitogenomes (Hugaboom *et al.* 2021). The complete mitochondrial genome for *Aspergillus fumigatus* SGAir0713 (GenBank accession: CM16889.1) was used as a reference for the seed database (parameter -s) for mitogenome assembly. Contigs generated for each *Aspergillus* species were circularized such that there was no overlap in the beginning and end of the mitochondrial genome sequence.

### Read mapping

Read mapping to correct errors was carried out using Bowtie2 v2.3.4.1 (Langmead and Salzberg 2012) and SAMtools v1.6 (Li *et al.* 2009). Bowtie2 aligned the raw paired-end reads from each *Aspergillus* species against the corresponding circularized mitochondrial genome. Variants were identified using SAMtools. Read mapping was also visualized and variants identified using the Integrative Genomics Viewer v2.9.4 (Robinson *et al.* 2017).

### Mitogenome annotation

The rapid organellar genome annotation software GeSeq v2.03 (Tillich *et al.* 2017) was used to annotate the circularized mitochondrial genomes. In addition to the newly assembled mitogenomes, mitochondrial reference genomes for *A. oryzae* and *A. sojae* were also annotated using GeSeq. Gene names were adjusted, and translations were checked in accordance with the reference mitochondrial genomes of *A. flavus* TCM2014 (NC_026920.1), *A. oryzae* 3.042 (NC_018100.1), *Aspergillus parasiticus* (NC_041445.1), and *A. fumigatus* A1163 (NC_017016.1). For *rnl* genes, GeSeq output was adjusted in accordance with both manual inspection in comparison to the above reference mitogenomes and NCBI Blast for similar sequences. Annotations were finalized following inspection of automated gene sequences using Geneious Prime v2021.1 (Kearse *et al.* 2012). OGDraw v1.1.1 (Greiner *et al.* 2019) was used for genome visualization.

### Multiple sequence alignment

Using MAFFT v7 (Katoh *et al.* 2019), single-gene multiple sequence alignment (MSA) files based on DNA nucleotide sequences were created for each of the 14 core mitogenes: cytochrome oxidase subunits 1, 2, and 3, NADH dehydrogenase subunits 1, 2, 3, 4, 4L, 5, and 6, ATP synthase subunits 6, 8, and 9, and cytochrome *b*. Gene nucleotide sequences corresponding to translated amino acid sequences for each gene were extracted from Geneious Prime v2021.1 (Kearse *et al.* 2012) sequence view and reverse complemented as necessary. The 14 individual MSA files were concatenated using SequenceMatrix v1.9 (Vaidya *et al.* 2011).

### Phylogenetic inference

To infer the evolutionary relationships within section *Flavi*, maximum likelihood phylogenies were constructed from both mitochondrial and nuclear data. The mitochondrial phylogeny was constructed from the MSA of 14 core concatenated mitogene nucleotide sequences files using RAxML v8.2.11 (Stamatakis 2014). The MSA was trimmed with ClipKIT v1.3.0 (Steenwyk *et al.* 2020) to retain parsimony-informative sites prior to construction of the phylogeny. *A. niger* (NC_007445.1) was used as the outgroup. We used 1,000 bootstrap replicates to evaluate robustness of inference. For the nuclear phylogeny, orthologous proteins in all species were identified using OrthoFinder v2.5.4 (Emms and Kelly 2019). MSAs for each of 2,422 orthologs were concatenated using the script catfasta2phyml.pl (https://github.com/nylander/catfasta2phyml, last accessed 2022 Apr). The maximum likelihood nuclear phylogeny was constructed with 1,000 replicates for bootstrapping using RAxML v8.2.11 (Stamatakis 2014) from the aligned orthologs shared among all the *Aspergillus* species in the study (including *A. niger*) except *A. sojae*, which did not have available sequencing data for nuclear genome assembly and annotation. For both nuclear and mitochondrial phylogenies, GTR + Γ substitution models were used in accordance with model testing performed within the raxmlGUI 2.0 platform (Kozlov *et al.* 2019; Edler *et al.* 2021). The resulting consensus trees for both the mitochondrial and nuclear phylogenies were visualized using Geneious Prime v2020.1.2 (Kearse *et al.* 2012).

### Cluster analysis

To compare patterns of synonymous codon usage bias between mitochondrial and nuclear genomes, hierarchical clustering of genome-level relative synonymous codon usage (RSCU) values was calculated and visualized using RStudio v. 2021.09.1. RSCU is a commonly used metric for codon usage bias that reflects the observed frequency of a particular codon divided by its expected frequency if all synonymous codons were used equally (Sharp and Li 1986b). Genome-level RSCU values as well as RSCU values for each mitochondrial and nuclear gene were computed using DAMBE v7.3.5 (Xia 2017).

### Correspondence analysis

To determine which codons drive differences in signatures of codon usage between nuclear and mitochondrial genes and between the mitogenomes of the 20 *Flavi* species, correspondence analyses were performed using gene-level RSCU values. Correspondence analysis (CA) was used for multivariate analysis because the RSCU values are interdependent—the RSCU values for 1 codon are inherently linked to the RSCU values of other synonymous codons—and thus not suited for principal component analysis. The correspondence analyses were carried out in RStudio v. 2021.09.1. using the packages ade4 v.1.7-19 (https://CRAN.R-project.org/package=ade4, last accessed 2022 May) and factoextra v.1.0.7 (https://CRAN.R-project.org/package=factoextra, last accessed 2022 May).

### Evaluation of mutational bias and codon usage

To evaluate the role of mutational bias in determining the observed patterns of codon usage bias in section *Flavi*, we plotted the effective number of codons (ENc) for each gene against their respective GC3 values, where GC3 is the GC content of the third codon position. ENc is often used to assess the nonuniformity of synonymous codon usage within individual genes (Wright 1990). Values range from 20 (extreme bias where only 1 codon is used per amino acid) to 61 (no bias). The ENc values for each gene were computed in DAMBE v.7.3.5 (Xia 2017). The resulting distribution was compared to the predicted neutral distribution proposed by dos Reis *et al.* (2004) using the suggested parameters by computing the *R*^2^ values between the observed and expected ENc values.

### Evaluation of selection on codon usage

To compare the influence of translational selection on the codon usage bias of mitogenomes as compared to nuclear genomes, we calculated the *S*-value proposed by dos Reis *et al.* (2004) for each species. The *S*-value is the correlation between the tRNA adaptation index (stAI) and the confounded effects of selection on the codon usage of a gene as well as of other factors (e.g. mutation bias, genetic drift). Therefore, the *S*-value measures the proportion of the variance in codon bias that cannot be accounted for without invoking translational selection. Thus, the higher the *S*-value, the stronger the action of translational selection on the given set of genes.

To calculate the *S*-value, we first measured tRNA counts for each nuclear and mitochondrial genome using tRNAscan-SE 2.0 (Chan *et al.* 2021). These counts were used to calculate the species-specific value for each codon’s relative adaptiveness (wi) in stAIcalc, version 1.0 (Sabi *et al.* 2017). Exclusively mitochondrial tRNA counts were used to obtain wi values for mitogenomes, whereas exclusively nuclear genome tRNA counts were used for nuclear genomes. Taking the geometric mean of all wi values for the codons yielded the stAI of each gene. These stAI values were then used to calculate *S*-values for each mitochondrial and nuclear genome with the R package tAI.R, version 0.2 (https://github.com/mariodosreis/tai, last accessed 2022 Apr).

The statistical significance of each *S*-value was tested via a permutation test. A total of 100 permutations were run such that each genome’s wi values were randomly assigned to codons, the tAI values recalculated for each gene, and the *S*-test run on that permutation. A genome’s observed *S*-value was considered statistically significant if it fell in the top 5% of the distribution formed by the 100 values obtained by the permutation analysis.

## Results

### Genomic content varies greatly between nuclear and mitochondrial genomes

**Fig. 1.**
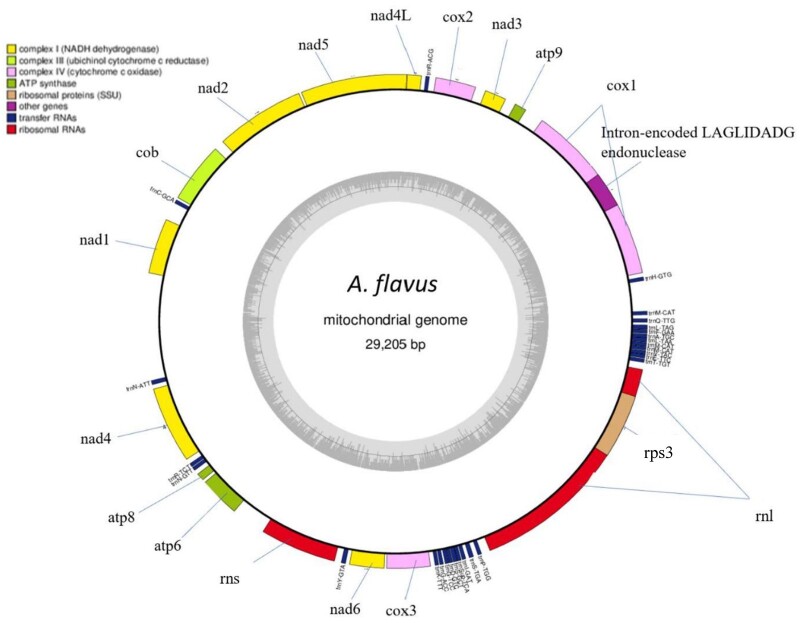
The typical *Aspergillus* section *Flavi* mitogenome is a circular DNA molecule. Here, the circularized mitogenome of *Aspergillus flavus* NRRL 1957 is visualized. The blocks around the outer circle indicate genes color coded by function. Each assembled section *Flavi* mitogenome shared a conserved set of 14 core mitochondrial genes, 2 rRNA genes, and 25–27 tRNA genes in the order pictured above. GC content (26.2% overall) is illustrated as the interior circle’s gray region.

**Fig. 2.**
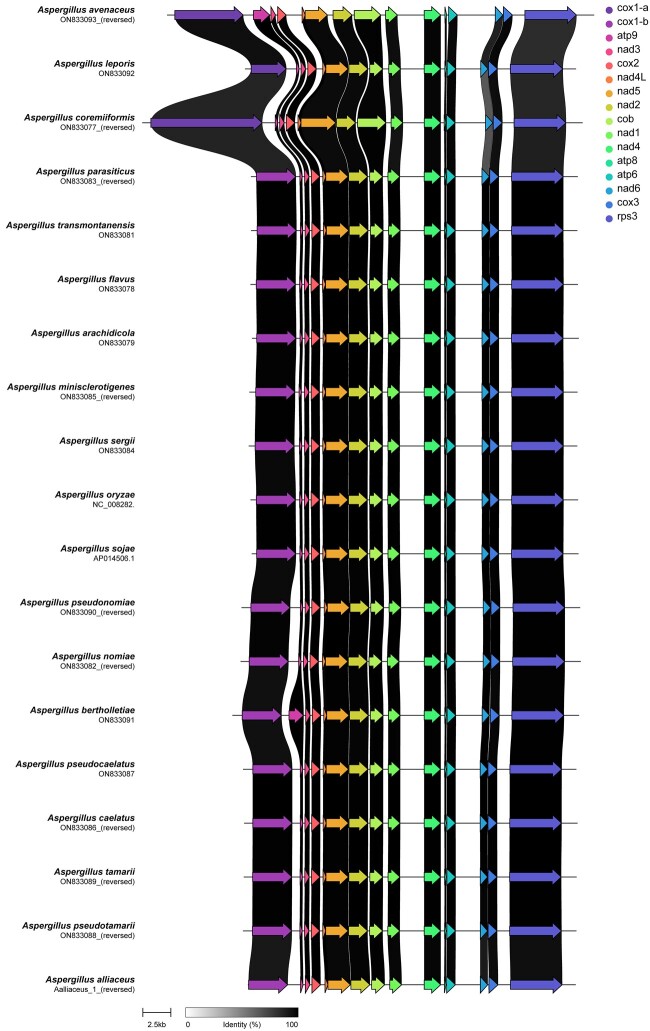
*Aspergillus* section *Flavi* mitogenomes demonstrate conserved gene content and order. Synteny plot of *Aspergillus* section *Flavi* mitochondrial protein-coding genes. Core mitochondrial genes are universally present in section *Flavi*, with conserved order. Each arrow represents a separate gene. Each line of arrows represents a different species. Connections between species illustrate nucleotide sequence conservation, with darker connections indicating higher similarity.

All mitogenomes were found to be small, circular DNA molecules with low GC content of 24.9–26.9% (Table 2 and Fig. 1). Each mitogenome contained 14 core genes (cytochrome oxidase subunits 1, 2, and 3, NADH dehydrogenase subunits 1, 2, 3, 4, 4L, 5, and 6, ATP synthase subunits 6, 8, and 9, and cytochrome *b*) with conserved order and shared synteny (Fig. 2). In addition, a ribosomal protein S3 was found in all newly annotated *Flavi* genomes, and an intron encoded LAGLIDADG endonuclease was found in all mitogenomes except for *Aspergillus avenaceus* and *Aspergillus leporis.* Variations in mitogenome length are due to variations in intron number and length, primarily in the *cox1* gene. Introns were universally present in the *cox1* gene, with most mitogenomes housing a single intron ranging from 1,393 to 1,780 bp. Two exceptions—*A. avenaceus* and *Aspergillus coremiiformis*—housed 3 and 4 introns of total length 4,344 and 3,504 bp, respectively, in their *cox1* genes. All mitogenomes also housed a single intron in their *rnl* gene ranging from 1,682 to 1,709 bp. Finally, *A. avenaceus* and *A. coremiiformis* were found to have additional introns. *A. avenaceus* has a 1,227-bp intron in its *atp9* gene and a 1,200-bp intron in its *cob* gene, while *A. coremiiformis* has a 1,104-bp intron in its *nad5* gene and a 1,379-bp intron in its *cob* gene.

**Table 2.** Mitochondrial and nuclear *Aspergillus* genomes differ greatly in size, genomic content, and GC bias.

	Genome length (kbp)	rRNAs	tRNAs	CDS	GC%
Mitogenomes	29.100–39.269	2	26	15–17	24.9–26.9
Nuclear genomes	30,1001–40,900	Undetermined	228–272	9,078–14,216	43–48.8

The summary above includes ranges of values from 20 *Aspergillus* section *Flavi* species’ mitochondrial and corresponding nuclear genomes.

Conversely, corresponding nuclear genomes are linear and have less extreme GC content biases ranging from 43.0 to 48.8% (Table 2). Nuclear genomes are roughly 1,000 times larger than their mitochondrial counterparts; while mitogenomes ranged from 29.100 to 39.269 kbp, nuclear genomes ranged from 30,1001 to 40,900 kbp. Of note, both nuclear and mitochondrial genomes house their own set of tRNAs (i.e. have their own tRNAome), although the tRNAome of nuclear genomes is roughly 10 times larger than that of mitochondrial genomes. While nuclear genomes house 228–272 tRNAs, the mitogenomes encode a conserved set of 26 tRNAs. Importantly, each amino acid is represented by at least 1 tRNA in the conserved mitochondrial tRNAome. However, the codons GCC, GCU, CGG, CUC, CUU, CCC, CCU, UCC, UCG, UCU, ACC, ACU, GUC, GUU, and UGG could not be decoded without invoking additional wobble hypotheses, modification of mitochondrial tRNAs or importation of nuclear tRNAs.

### Mitochondrial and nuclear phylogenies are very similar

**Fig. 3.**
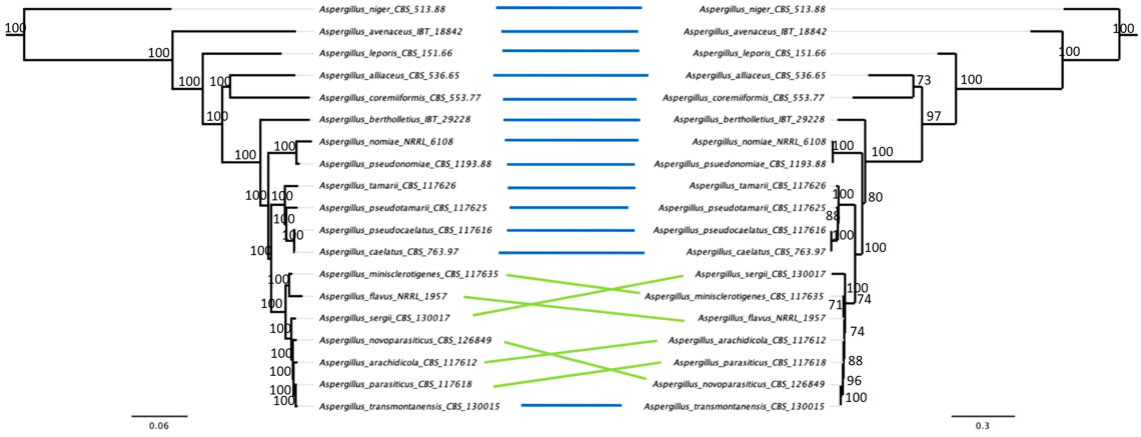
Phylogenies constructed from nuclear and mitochondrial data predict similar evolutionary relationships, with minor differences in inferred topology arising amongst more closely related species. a) Maximum likelihood phylogeny based on concatenation of 2,422 nuclear orthologs with bootstrap values from 1,000 replicates. b) Maximum likelihood phylogeny based on concatenation of 14 core mitogene sequences with bootstrap values from 1,000 replicates. Numbers above nodes indicate bootstrap values.

To understand how the evolutionary history of section *Flavi* informed by mitogenomes compares to that of nuclear genomes, a mitochondrial phylogeny was constructed using a concatenation of 14 core mitogene nucleotide sequences (Fig. 3b). The resulting phylogeny displayed high bootstrap support. Despite minor topological differences from a well-supported nuclear phylogeny (Fig. 3a) among more closely related species, the evolutionary relationships predicted by the mitochondrial phylogeny largely align with those predicted by the nuclear phylogeny. For instance, although *Aspergillus minisclerotigenes*, *Aspergillus sergii*, *A. flavus*, *Aspergillus arachidicola*, *A. parasiticus*, and *Aspergillus novoparasiticus* fall within the same clade in both nuclear and mitochondrial phylogenies, the predicted evolutionary relationships within this clade vary slightly. Evolutionary rate was found to be more rapid in mitochondrial genomes relative to nuclear genomes. For example, the evolutionary distance between *A. flavus* and *A. nomiae* was 0.086 substitutions per site in the nuclear phylogeny, but 0.244 substitutions per site in the mitochondrial phylogeny. Single-gene mitochondrial phylogenies differed in their topologies but exhibited low bootstrap support values, particularly for relationships among closely related species (Supplementary File 1).

### Species groupings based on patterns of codon usage bias differ between mitochondrial and nuclear genomes

**Fig. 4.**
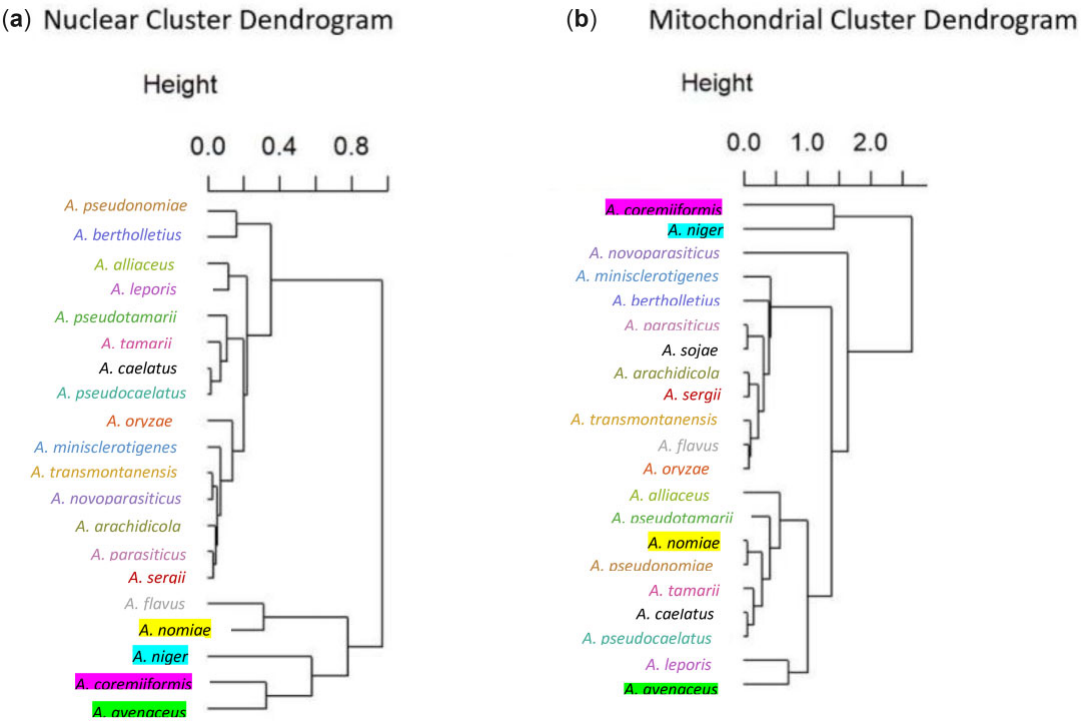
Hierarchical clustering analyses of RSCU values of mitochondrial and nuclear protein-coding regions demonstrate different species groupings based on mitochondrial and nuclear data. Dendrograms are colored/highlighted by species. a) Cluster analysis based on net RSCU values of nuclear protein-coding genes. b) Cluster analysis based on net RSCU values of mitochondrial protein-coding genes.

To compare similarities in interspecies patterns of CUB between mitochondrial and nuclear genomes, hierarchical clustering was performed using the net RSCU values of protein-coding regions of both nuclear (Fig. 4a) and mitochondrial (Fig. 4b) genomes. The cluster analyses predict different interspecies relationships depending on organelle of genomic origin. For example, the cluster dendrograms show that *A. coremiiformis* and *A. avenaceus* cluster together based on patterns of nuclear CUB, but group in completely different clusters based on mitochondrial CUB. This suggests that different pressures may govern CUB in mitochondrial genomes than in their nuclear counterparts.

### Patterns of codon usage bias reflect whether genes are mitochondrial or nuclear in origin

**Fig. 5.**
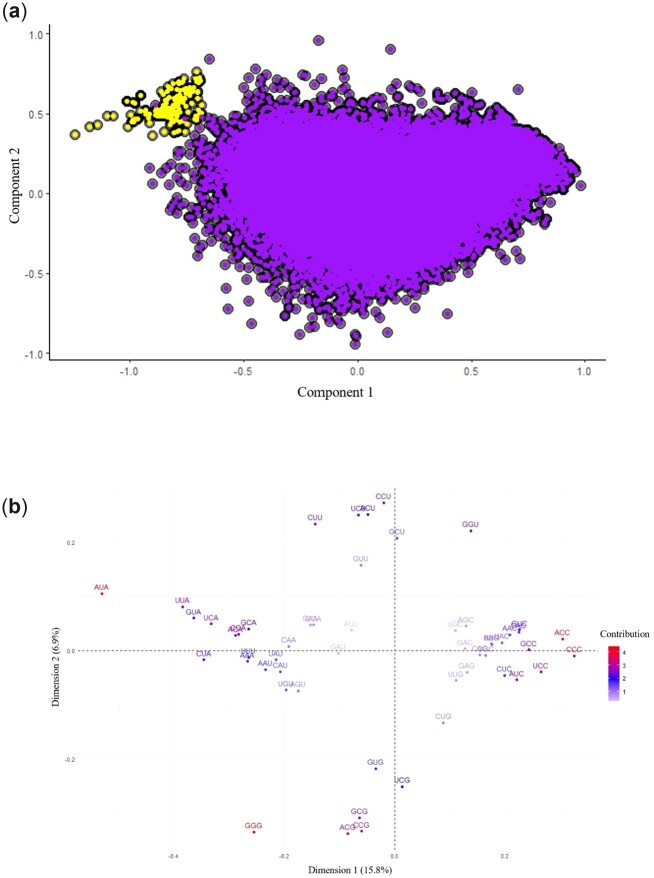
Correspondence analysis based on relative synonymous codon usage values reveals that signatures of codon usage bias are more similar based on organelle of origin as opposed to species of origin. a) Correspondence analysis plot of all mitochondrial (yellow/lighter dots in upper left) and nuclear (purple/darker dots) protein-coding genes for 20 *Aspergillus* section *Flavi* species. Each dot represents a gene. b) Factor map of codon contributions. Location of genes in correspondence analysis plot is driven largely by the GC content in the third position of synonymous codons used in the gene of interest.

To examine signatures of codon usage between nuclear and mitochondrial genes, RSCU values for each gene in each available genome were calculated. A CA was then performed to determine which codons drive observed differences in codon usage patterns (Fig. 5). The CA plot (Fig. 5a) shows a distinct clustering of the majority of the mitogenes away from nuclear genes. This demonstrates that codon usage signatures depend more on whether genes are mitochondrial vs nuclear as opposed to whether genes belong to the same species. The factor map of codon contributions (Fig. 5b) revealed that the first dimension explains 15.6% of observed variation between genes in the final plot. The second dimension explains 7% of observed variance. Examining dimensional contributions by codon reveals that the GC content of the third position drives separation along dimensions. Position along the first dimension (X-axis) is driven primarily by the usage of NNA vs NNC codons. The largest contributions along the X-axis come from the usage of AUA (isoleucine) and CCC (proline). RSCU values of greater than 1 indicate that a codon is overrepresented within a given synonymous codon group whereas RSCU values less than 1 indicate underrepresentation. The average RSCU of AUA and CCC are 1.6304 and 0.0449 in the mitochondria and 0.4525 and 1.0457 in the nucleus, respectively. Position along the second dimension (Y-axis) is driven primarily by differences in the usage of NNU vs NNG codons. The largest contributions along the Y-axis are from CCU (proline) vs GGG (glycine), ACG (threonine), and CCG (proline) combined. The average RSCU of CCU, GGG, ACG, and CCG are 2.7140, 0.0558, 0.0204, and 0.0484 in the mitochondria and 1.072, 0.7240, 0.8274, and 0.8920 in the nucleus, respectively.

**Fig. 6.**
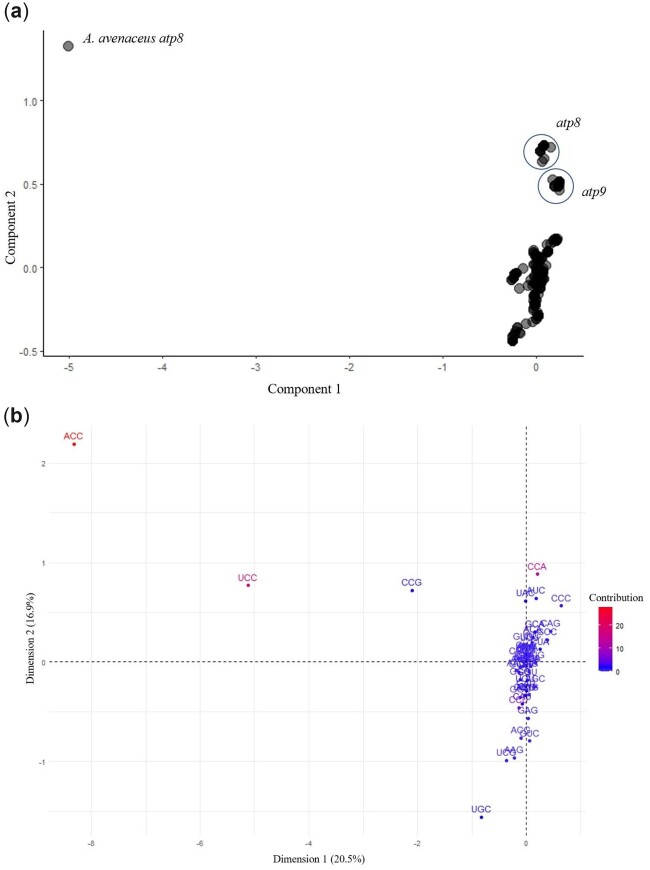
Correspondence analysis based on relative synonymous codon usage values in mitogenes reveals that signatures of codon usage bias are more similar based on gene identity as opposed to species of origin. a) Correspondence analysis plot of all mitochondrial protein-coding genes for 20 *Aspergillus* section *Flavi* species. Labels correspond to gene identity. b) Factor map of codon contributions

A second CA was run using the RSCU values for each mitogene to determine which codons drives observed interspecies differences in codon usage patterns in mitogenomes (Fig. 6). The CA plot shows distinct grouping based on gene identity as opposed to species of origin (Fig. 6a). The factor map of codon contributions revealed that the first dimension explains 18.7% of observed variation in the final CA plot, while the second-dimension accounts for 16.1% (Fig. 6b). The *A. avenaceus atp8* gene is a clear outlier along both axes. The codons that contribute the most to this are ACC, UCC, and CCG which are used at a frequency of 4, 4, and 1.33, respectively, in this gene. RSCU values of 4 indicate that only ACC (threonine) and UCC (serine)—none of the other synonymous codons within their respective families—are used in this gene. This degree of bias is expected given that threonine and serine occur only once and twice, respectively, in *A. avenaceus atp8.*

### Deviation of gene-level codon usage from neutral expectation varies based on whether genes are of nuclear or mitochondrial origin

**Fig. 7.**
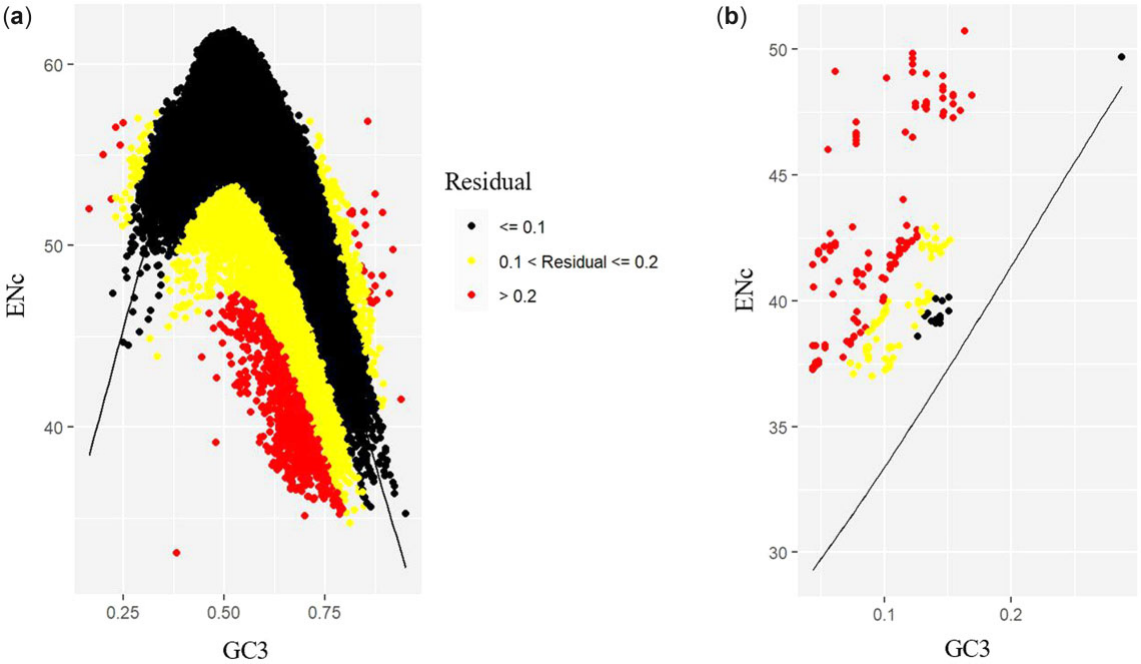
Most signatures of codon usage bias in mitochondrial and nuclear genes in *Aspergillus* section *Flavi* deviate from the expected codon usage bias under mutation pressure alone. a) ENc-GC3 plot for all nuclear protein-coding genes of 20 *Aspergillus* section Flavi genes plotted against the predicted neutral distribution. *R*^2^ value of 0.598 indicates moderate fit to neutral expectation. b) ENc-GC3 plot for all protein-coding mitogenes. *R*^2^ value of 0.211 indicates poor fit to neutral expectation.

To assess the role of mutational bias across all mitochondrial and nuclear genes, we examined the relationship between the ENc of each gene and its GC3 content by comparing observed ENc values to the expected relationship between ENC and GC3 content if codon usage was influenced by neutral mutational bias alone. We tested the fit to the neutral expectation of the complete dataset of all species’ combined nuclear and mitochondrial gene datasets as well as all nuclear genes and all mitogenes separately by calculating the *R*^2^ value. For all 20 species, combined nuclear and mitochondrial datasets yielded *R*^2^ values greater than 0.5, suggesting that codon usage in these species can be partially explained by neutral mutational bias (Supplementary File 2). Furthermore, patterns of deviation from the neutral expectation were highly similar between species (Supplementary File 2). However, when nuclear and mitochondrial genes were analyzed separately, nuclear genes had an *R*^2^ value of 0.598, whereas mitochondrial genes had an *R*^2^ value of 0.211 (Fig. 7). This suggests that, although codon usage in nuclear genomes can be partially explained by neutral mutational bias, mutational bias does not fully account for the codon bias in mitochondrial genomes.

### Codon usage in nuclear genomes, but not mitogenomes, is under translational selection

**Fig. 8.**
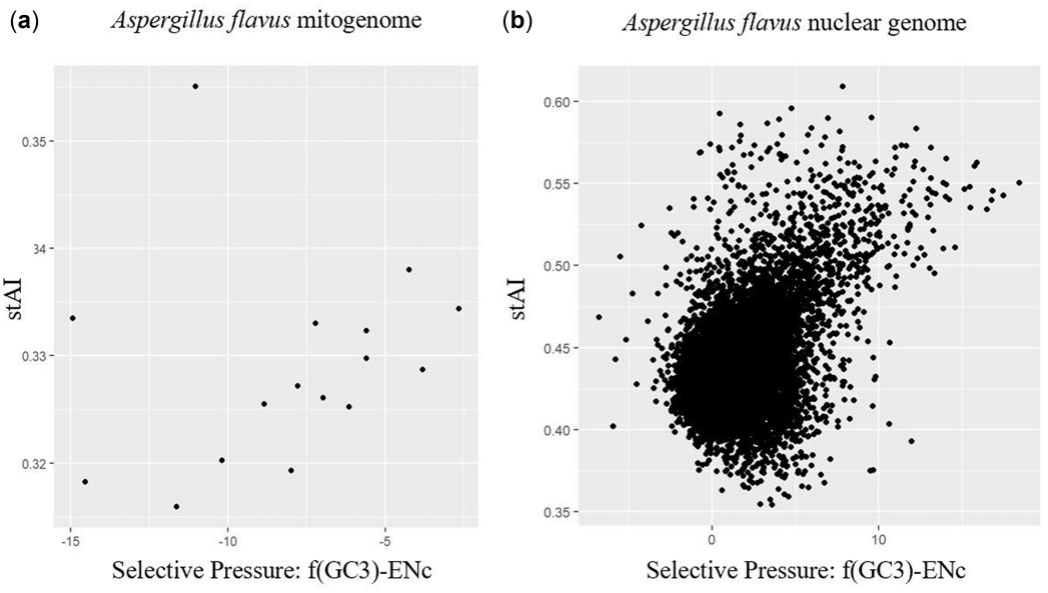
Section *Flavi* mitogenomes are not under significant translational selection on codon usage bias, but nuclear genomes display moderate translational selection. Plots of stAI against selective pressure for all protein-coding genes of *Aspergillus flavus*. a) Mitogenes only. Example of insignificant translational selection on *S*-test (*S* = 0.191). b) Nuclear genes only. Example of moderate translational selection on *S*-test (*S* = 0.454).

To test if translational selection could account for the observed deviations of CUB from the neutral expectation, we calculated the *S*-values for each mitochondrial and nuclear genome. Of the 20 *Flavi* species tested, mitogenome *S*-values ranged from −0.103 to 0.392 with a median value of 0.162 and mean value of 0.137 (Fig. 8a). However, no species’ mitogenomes had *S*-values that were found to be significant in the permutation test. In contrast, nuclear genome *S*-values ranged from 0.269 to 0.502, with a median value of 0.432 and a mean value of 0.427 (Fig. 8b). The *S*-value of *A. novoparasiticus* (*S* = 0.269) was calculated using the package tAI.R (https://github.com/mariodosreis/tai/blob/master/R/tAI.R, last accessed 2022 Apr). This was done as the original calculation with stAI calc created issues with file merging. All nuclear *S*-values were found to be significant in the permutation test, suggesting that *Flavi* nuclear genomes are under moderate levels of translational selection.

## Discussion

In this study, we compared the evolution of mitochondrial and nuclear genomes within *Aspergillus* section *Flavi*. We assembled and annotated the mitogenomes of 18 *Flavi* species and reannotated 2 previously assembled reference mitogenomes. We then used phylogenetic analyses to compare phylogenies derived from nuclear vs mitochondrial data. Finally, we examined the patterns of and forces underlying CUB in nuclear and mitochondrial genomes.

The newly assembled mitogenomes are comparable in gene content and size to previously published *Aspergillus* mitogenomes. At 29.10–39.27 kb, the range of *Aspergillus* section *Flavi* mitogenome length falls within the lower range of published fungal mitogenomes, which vary in size from 12.06 to 235.85 kb (Joardar *et al.* 2012; Zhang *et al.* 2020). GC content was consistent with low percentages observed in other *Aspergillus* and related fungal species (Machida *et al.* 2005; Sato *et al.* 2011; Joardar *et al.* 2012; Zhao *et al.* 2012; Yan *et al.* 2016; Park *et al.* 2019, 2020; Hugaboom *et al.* 2021). The mitogenomic content and gene order were highly conserved in the 20 *Flavi* species analyzed, and all mitogenomes examined contained 14 core mitochondrial genes. As in previous studies, these core genes showed high levels of sequence similarity and conservation of gene order (Joardar *et al.* 2012; Hugaboom *et al.* 2021). Fungal mitogenomes are also known to contain accessory genes in addition to the core set of 14. The presence of the 2 accessory genes—an intron encoded LAGLIDADG endonuclease and the ribosomal protein S3—in most of the species analyzed is also consistent with existing *Flavi* annotations (Joardar *et al.* 2012; Hugaboom *et al.* 2021). The order of these accessory genes was also highly conserved. Of note, the mitogenomes contained their own set of 26 tRNAs separate from the nuclear-encoded set of tRNAs. In analyses of CUB, the mitochondrial tRNAs were used to determine if mitochondrial CUB patterns had been optimized to the mitochondrial tRNA pool.

In comparing the topologies and evolutionary rates predicted by phylogenies derived from nuclear and mitochondrial data, we found that their inferred evolutionary histories were similar. The high degree of congruence in the 2 phylogenies suggests potential coevolution of mitochondrial and nuclear genes. The minor disagreements between the 2 phylogenies may be explained by phenomena that occur uniquely in mitochondria. For example, fungal mitochondria are uniparentally inherited (Santamaria *et al.* 2009; Horn 2016). In addition, fungal species can undergo interspecific hybridization (Giordano *et al.* 2018). In this process, the mitochondria of 1 species may be inherited by the other. Moreover, mitochondrial recombination events with repeated backcrossing can lead to introgression (Giordano *et al.* 2018). Interspecific introgression and recombination occur in fungal nuclei as well. Thus, the 2 phylogenies may differ due to disparities in interspecific introgression and/or recombination occurring in the mitochondria or nuclei of section *Flavi.* Alternatively, however, topological differences could arise due to sampling error, as mitochondrial genes contain few sites relative to nuclear genes, for example.

Cluster analyses based on net RSCU values demonstrated that interspecies similarities in patterns of CUB differ between nuclear and mitochondrial genomes. Despite some parallels in predicted grouping—for example, the grouping of *A. caelatus* and *A. pseudocaelatus* in both dendrograms—mitochondrial and nuclear cluster analyses displayed groupings largely inconsistent with each other. It is important to note that all the species included in this study have similar mean values of codon usage metrics (ENc, GC content, and GC3s) within the nuclear and mitochondrial genome. Thus, well-resolved interspecies relationships are unlikely to be based on codon usage indices alone. Alternatively, the observed incongruence may reflect different pressures governing CUB in mitochondrial compared to nuclear genomes.

Examination of codon usage patterns using correspondence analyses showed that differential usage of certain codons drives observable differences in signatures of CUB between mitochondrial and nuclear genes and between gene type in mitogenes. Differential usage of specific codons between nuclear and mitochondrial genomes appears to rely heavily on the GC content of the third position of synonymous codons. This pattern aligns with overall GC content of the genomes. For example, the use of the codon AUA contributes to the placement of the mitogenomes in quadrant II of the final CA plot, where mitogenes tend to cluster, while the use of AUC contributes to the placement of the nuclear genes in quadrants I and IV. The average RSCU values of AUA and AUC are 1.6304 and 0.1782 in the mitochondria and 0.4525 and 1.4760 in the nucleus, respectively. Both of these codons code for isoleucine, yet mitogenes are enriched for the AUA codon and nuclear genes for the AUC codon, as would be expected based on the differences in GC content between the 2 genomes. The separation of mitogenes is also dependent on the GC content of the third position. Fig. 6 shows that, while the use of most codons is similar among all mitogenes, the occurrence of a rare G- or C-ending codon drives separation based on CUB patterns. This is especially clear in the case of the outlying *A. avenaceus atp8* gene in Fig. 6a, which is driven by the higher use of codons ACC, UCC, and CCG. Despite a high degree of sequence conservation with the other 19 *atp8* nucleotide sequences (Supplementary File 3), the change in a small number of nucleotides at third codon positions results in a large visible separation in the CA plot (Fig. 6a). This effect is amplified due to the short, highly conserved nature of the *atp8* gene sequences. Overall, we found that gene-level RSCU values allow for observable differences in CUB pattern based on the organelle of genomic origin and mitogene identity.

We also sought to determine the relative importance of neutral processes and natural selection on shaping CUB in mitochondrial and nuclear genomes. Based on ENc-GC3 plots, most mitogenes fell at least 20% from the neutral expectation, while most nuclear genomes fell within 10% of the neutral expectation. These results reinforce previous findings that CUB varies at the gene level within a species (Sharp *et al.* 1988; Chen *et al.* 2004; LaBella *et al.* 2019). Of note, studies have shown that greater divergence from the neutral expectation is moderately associated with increased expression (Tsankov *et al.* 2010). Future avenues may examine the association between the large residuals from the neutral expectation and expression levels of mitogenes.

The moderate to poor fit to the neutral expectation for nuclear and mitochondrial genes, respectively, suggests that mutational bias alone cannot account for the observed patterns in codon usage bias. By using the *S*-test to test for the influence of translational selection, we found that gene-level codon usage in mitochondrial genomes could not be significantly distinguished from neutral mutational bias—including both selectively neutral changes and purifying selection—in section *Flavi*, while translational selection acts moderately on codon usage bias in nuclear genomes. The lack of significant translational selection on mitogenomes is unsurprising, given their extreme GC bias and small size. This may be a manifestation of mtDNA evolving clonally with limited ability to recombine; thus, CUB is more likely to reflect mutation bias and drift rather than selection. The faster evolutionary rates of mitogenomes may lead to genetic drift playing a larger role in shaping CUB than in corresponding nuclear genomes. In addition, when genome size is small, it is hypothesized that low tRNA redundancy limits the ability of selection to act on CUB (dos Reis *et al.* 2004). Of note, *S*-value calculation for mitogenes was limited to a dataset of 16 genes. Visual inspection of the data used to determine the *S*-values suggests a general positive correlation between selective pressure and codon usage—which would suggest translational selection on codon usage—that is obscured by a couple outlier genes (Fig. 8a). This observation in combination with the highly variable codon usage between mitochondrial genes suggests that the balance between selective and neutral forces on mitochondrial codon usage may vary greatly between mitogenes. Finally, the final *S*-value calculations for mitogenomes were based solely on the mitochondrial tRNA counts derived from genomic sequences and not experimental tRNA abundances. In fact, our analysis suggests that additional tRNA dynamics, such as modification or importation, may be at work in *Aspergillus* mitochondria.

Computational analysis of codon usage and tRNA composition in *Aspergillus* mitogenomes suggests that there is a significant gap in our knowledge of tRNA dynamics within these organelles. It is known that mitochondria can employ diverse strategies to obtain a complete and functional set of tRNAs; some organisms such as the fungus *Saccharomyces cerevisiae* encode a complete set of tRNA genes within their mitochondria (Salinas-Giegé *et al.* 2015) while others require the importation of nuclear tRNAs into the mitochondria (Alfonzo and Söll 2009). Our analysis demonstrates that, 15 codons cannot be decoded by the mitochondrial tRNAome without invoking liberal wobble base pairing, mitochondrial tRNA modification, or import of nuclear tRNAs (Supplementary File 4). This suggests that tRNA import or modification may be occurring in *Aspergillus* mitochondria. In addition, mitochondrial codon usage is not consistently biased toward codons matching the mitochondrial tRNAome. For example, the codon GCA (alanine), which can be decoded by a mitochondrial tRNA, has an average RSCU value of 1.4222 in mitogenomes, whereas the codon GCU (also alanine) has an average RSCU value of 2.3423 even though the mitochondrial tRNAome is unable to decode this codon. The preference for GCU codons suggests the importation or modification of a tRNA capable of decoding this codon. Finally, the *Aspergillus* mitochondrial tRNAs fit the wobble versatility hypothesis for each codon family, with the exception of CGN (arginine), UGR (tryptophan), and AUR (methionine), a finding that is consistent with previous investigation of the wobble nucleotide position in fungal mitogenomes (Supplementary File 5) (Carullo and Xia 2008). That is, the anticodons of the mitochondrial tRNAome have nucleotides at the wobble site that maximize versatility in wobble base pairing as opposed to maximizing Watson-Crick base pairing with the most frequently used codon within each synonymous codon family. Improving our understanding of *Aspergillus* mitochondrial tRNA dynamics will not only allow us to better understand translational dynamics within the organelle but recent work has suggested that mitochondrial tRNAs may play a role in antifungal response (Colabardini *et al.* 2022).

Despite a limited understanding of tRNA dynamics within *Aspergillus* mitochondria, our results are consistent with the limited role of translation selection in shaping general patterns of mitochondrial codon usage in other species including budding yeasts, plants, and animals (Kamatani and Yamamoto 2007; Jia and Higgs 2008; Zhou and Li 2009). As with previous work, we also noted a few specific codons (proline codons) and genes (*atp8*) with increased biases that may be related to factors such as wobble-decoding or tRNA abundance.

In summary, analysis of mitochondrial and nuclear genome data from *Aspergillus* section *Flavi* revealed that both genomes are largely phylogenetically congruent and that the pattern and evolutionary forces shaping CUB differ between the mitochondrial and nuclear genomes. These evolutionary analyses, coupled with the generation of mitogenome assemblies for 18 section *Flavi* species, contribute to our understanding of genome evolution in the genus *Aspergillus.*

## Data Availability

The newly assembled *Aspergillus* section *Flavi* mitogenomes from this study are available in GenBank under accession no. ON833077, ON833078, ON833079, ON833081, ON833082, ON833083, ON833084, ON833085, ON833086, ON833087, ON833088, ON833089, ON833090, ON833091, ON833092, ON833093, and ON833094. Reannotations for previously assembled mitogenomes are available through figshare (10.6084/m9.figshare.20412186). The SRA accession numbers for whole genome sequencing data used for mitogenome assembly are provided in Table 1. For previously assembled mitogenomes, the NCBI reference sequence GenBank accession numbers are provided in place of SRA accession numbers. Additional data, including supplementary data, are available through figshare (https://doi.org/10.6084/m9.figshare.20412186).
